# The role of endothelin B receptor in bone modelling during orthodontic tooth movement: a study on ET_B_ knockout rats

**DOI:** 10.1038/s41598-020-71159-8

**Published:** 2020-08-26

**Authors:** S. Ibrahimi Disha, B. Furlani, G. Drevensek, A. Plut, M. Yanagisawa, S. Hudoklin, I. Prodan Žitnik, J. Marc, M. Drevensek

**Affiliations:** 1 Department of Orthodontics, Faculty of Medicine, University of Ljubljana, Hrvatski trg 6, 1000 Ljubljana, Slovenia; 2 Institute of Pharmacology and Experimental Toxicology, Faculty of Medicine, University of Ljubljana, Ljubljana, Slovenia; 3 Department of Orthodontics, University Medical Center Ljubljana, Ljubljana, Slovenia; 4 International Institute for Integrative Sleep Medicine, University of Tsukuba, Tsukuba, Japan; 5 Institute of Cell Biology, Faculty of Medicine, University of Ljubljana, Ljubljana, Slovenia; 6 Department of Clinical Biochemistry, Faculty of Pharmacy, University of Ljubljana, Ljubljana, Slovenia

**Keywords:** Medical research, Molecular medicine

## Abstract

The endothelin system has an important role in bone modelling during orthodontic tooth movement (OTM); however, little is known about the involvement of endothelin B receptors (ET_B_) in this process. The aim of this study was to evaluate the role of ET_B_ in bone modelling during OTM using ET_B_ knockout rats (ET_B_-KO). Thirty-two male rats were divided into 4 groups (n = 8 per group): the ET_B_-KO appliance group, ET_B_-KO control group, wild type (ET_B_-WT) appliance group, and ET_B_-WT control group. The appliance consisted of a super-elastic closed-coil spring placed between the first and second left maxillary molar and the incisors. Tooth movement was measured on days 0 and 35, and maxillary alveolar bone volume, osteoblast, and osteoclast volume were determined histomorphometrically on day 35 of OTM. Next, we determined the serum endothelin 1 (ET-1) level and gene expression levels of the osteoclast activity marker cathepsin K and osteoblast activity markers osteocalcin and dentin matrix acidic phosphoprotein 1 (DMP1) on day 35. The ET_B_-KO appliance group showed significantly lower osteoblast activity, diminished alveolar bone volume and less OTM than the ET_B_-WT appliance group. Our results showed that ET_B_ is involved in bone modelling in the late stage of OTM.

## Introduction

Endothelin 1 (ET-1) plays an important role in the regulation of bone metabolism in physiological as well as in pathophysiological processes^1–4^. It has a known role in the maintenance of bone homeostasis and the regulation of osteoblastic function. ET-1 stimulates the proliferation, differentiation and activity of osteoblasts^5,6^ and inhibits osteoblast apoptosis, promoting osteoblastic growth^7^. ET-1 acts through both endothelin A (ET_A_) and endothelin B (ET_B_) receptors. Specifically, it has been shown that the ET-1/ET_A_ axis is an important regulator of osteoblast activity; targeted inactivation of ET_A_ in mature osteoblasts induced lower tibial trabecular bone volume in vivo^8^. However, less is known about the role of ET_B_ in osteogenesis. In one study, treatment with the dual ET_A_ and ET_B_ antagonist Macitentan showed decreased vertebral bone mass in mice, potentially from decreased osteoblast activity as well as from the increased osteoclast activity^9^.

**Figure 1:**
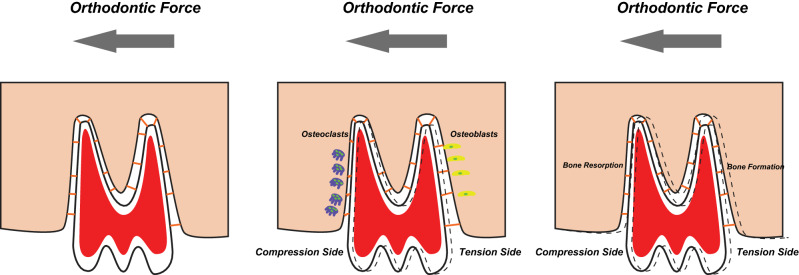
Model of the OTM. Applying of orthodontic force to the tooth compresses the PDL. At the compression side of the tooth, which appears in the direction of the orthodontic force, bone resorption takes place, carried out mainly by osteoclasts. At the tension side, osteoblasts are responsible for the bone formation process.

Several studies also indicate the involvement of endothelins during orthodontic tooth movement (OTM)^10–12^. Orthodontic movement is a consequence of applying a force to the teeth. It is a mechanism that involves the biomechanical adaptation of the alveolar process and supporting periodontium. Alterations in the vascularity within the periodontal ligament (PDL), the connective tissue that connects a tooth to its surrounding alveolar bone, may trigger responses at the cellular level, such as alveolar bone modelling^13,14^. During OTM areas of pressure and tension are formed in the PDL. At pressure areas, which appear in the direction of the application of force, alveolar bone is resorbed, and at tension areas, which appear on the opposite side, new bone is formed (Fig. 1). Therefore the animal model of OTM can be used to study the accelerated bone modelling^15,16^. OTM consists of three different phases—the initial, lag and late phase. In the late phase linear teeth movement can be observed^17^.

ET-1 release in the PDL begins after 3 h of continuous loading of a rat molar^18^. However, in the alveolar bone ET-1 gene expression levels increased for the following 4 weeks after the start of the application of orthodontic force and predominated in the late phase of OTM, when the ET_B_ expression rate was also upregulated^11^. A selective ET_A_ antagonist significantly increased alveolar bone volume and decreased osteoclast volume and the amount of OTM, indicating decreased bone resorption in the late stage of OTM, and confirming the role of ET_A_ in accelerated bone modelling^11^. On the other hand, a dual-selective endothelin antagonist (ET_A_/ET_B_) increased the amount of OTM in rats after 25 days of treatment^12^. Furthermore, the gene expression levels of both ET_A_ and ET_B_ were increased in the late phase of OTM^11^, when bone formation on the tension side and bone resorption on the pressure side of the loaded tooth were reported^17^, suggesting that both receptor subtypes could be involved in the process of the late stage of OTM.

The multipotent stem cells in the alveolar bone marrow, the PDL and the periosteum all participate in the regulation of bone remodelling and tooth movement. Mesenchymal stem cells (MSCs), when stimulated by a mechanical strain, differentiate into an osteo-chondrogenic lineage with increased expression of osteoblastic and chondrogenic markers^19^. Periodontal ligament stem cells (PDLSCs) are tissue-specific MSCs in PDL that play an important role during OTM^20^. It has been shown that both tension and compression can regulate the osteogenic differentiation of PDLSCs^16,21^. Interestingly, it has also been reported that ET-1 promotes the osteogenic differentiation of bone marrow-derived MSCs^22^ and PDLSCs^23^. Moreover, some studies mention an important role of both ET_A_ and ET_B_ receptors in the differentiation of different types of MSCs into different cellular phenotypes^24,25^.

ET-1 has been shown to have anti-apoptotic activity in numerous tissues. For example, it inhibits bone degradation by the induction of anti-apoptotic activity, and stimulates bone formation via endothelin ET_A_ receptors in vitro and in vivo^26^. ET-1 is also known as an anti-apoptotic factor in osteoblasts^7^, and it promotes osteosarcoma cell invasion and survival against cisplatin-induced apoptosis through the ET_A_ receptor^4^. The anti-apoptotic effects of ET-1 have been described in several other tissues: neurons^27^, cardiomyocytes^28^, and airway smooth muscle cells^29^.

The role of ET_B_ has mostly been studied in cancer cell lines and in cancer tissue cultures^30^. The activation of ET_B_ receptors by ET-1 has been shown to affect the processes involved in the inhibition of cancer, inducing cell death by apoptosis and promoting ET-1 clearance^31,32^. ET_B_ expression has also been reported in many tumour types, including prostate cancer^33,34^ melanomas^35^, and oligodendrogliomas^36^. For example, in prostate cancer, the downregulation of ET_B_ results in a higher local concentration of ET-1 which, through the stimulation of ET_A_ receptors, facilitates cancer progression, including proliferation, escape from apoptosis and new bone formation^34^. ET_B_ has been predominantly classified as a “clearance receptor”, and the role of circulatory ET-1 clearance by ET_B_ has been confirmed in several studies^32,37,38^. In healthy men an injection of a selective ET_B_ antagonist increased the plasma concentration of ET-1 and confirmed the crucial role of ET_B_ in the clearance of endothelins in humans^39^.

Despite the fact that the role of ET_A_ in bone modelling during OTM has been studied, the role of ET_B_ in bone modelling in OTM is not well understood at present. The aim of this study was to determine the role of ET_B_ in bone modelling in OTM using ET_B_–KO rats and to evaluate its effect on osteoclastogenesis and osteoblastogenesis in comparison to bone modelling in ET_B_–WT rats.

## Material and methods

### Laboratory animal model

The study was performed on ET_B_ knockout (ET_B_-KO −/−) and wild type (ET_B_-WT +/+) rats. The ET_B_-KO rat line and its control line ET_B_-WT was established at the laboratory of Masashi Yanagisawa, PhD, at Howard Hughes Medical Institute (University of Texas Southwestern Medical Centre, Dallas, Texas, USA). The ET_B_-KO animals were incorporated with a dopamine beta-hydroxylase (DβH) transgene to enable the development of a normal enteric nervous system. The DβH transgene was also inserted into the control animals^40^. The rats were bred in a homozygous line at the Faculty of Medicine, University of Ljubljana (Slovenia). In this study we used 32 male ET_B_-KO rats (285 ± 27 g, 13–15 weeks old) and 32 male ET_B_-WT rats (286 ± 30 g, 13–15 weeks old). The animals were housed as well as procedures were identical to our previous studies^11^. The part of daily intake of rat chow (Teklad 2016 Global rodent diet, Harlan, The Netherlands) was soaked in water to facilitate food intake due to its mild impairment during orthodontic force application.

To ensure general anaesthesia, the anaesthetics were injected intraperitoneally: ketamine 50 mg/kg body weight (Bioketan, Vetoquinol Biowet, Gorzów Wielkopolski, Poland), medetomidine hydrochloride 67 mg/kg body weight (Domitor, Pfizer, Brooklyn, NY, USA), and thiopental 3 mg/kg body weight (Tiopental, Pliva, Zagreb, Croatia)^12^.

All the animal procedures and the study protocol were approved by the Ethics Committee for Animal Experiments of the Administration of the Republic of Slovenia for Food Safety, Veterinary Sector and Plant Protection (34401-62/2008/20), and complied with the guiding principles in ''The Care and Use of Animals''.

## Study protocol

Thirty-two rats were divided into the following four groups: (1) ET_B_-KO appliance group (n = 8); (2) ET_B_-KO control group (n = 8); (3) ET_B_-WT appliance group (n = 8); (4) ET_B_-WT control group (n = 8).

**Figure 2:**
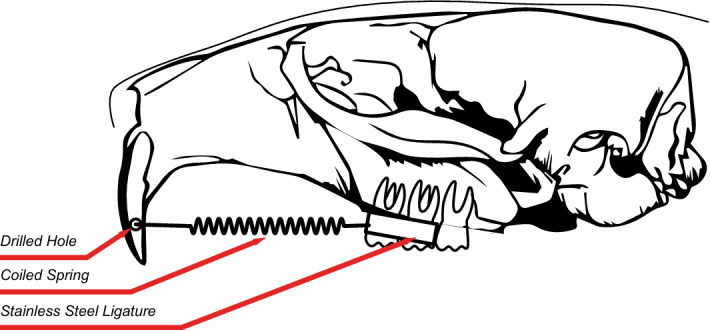
Schematic view of the orthodontic appliance.

The orthodontic appliance consisted of a superelastic closed coil spring (25 cN; wire diameter, 0.15 mm; GAC Dentsply International, York, PA, USA) which was placed between the first and second left maxillary molars and the incisors by a stainless-steel ligature in the ET_B_-KO and ET_B_-WT appliance groups^11^. The coil spring was attached to a steel thread placed around the first and second molar on one side and through a drilled hole into the upper incisors on the other side (Fig. 2). The hole was drilled laterally in the incisors through the area where the vivid tooth structures were unaffected^12^. The orthodontic appliance was placed in each animal under general anaesthesia at the beginning of the study, and replaced to the correct position every 7 days, ensuring its proper activation and the exertion of constant force on the teeth^41^.

### Tooth movement measurements

The distance between the most mesial point of the maxillary first molar and the most distal point of the ipsilateral incisor at the gingival level was measured on the experimental sides in all four groups. The measurements were obtained using a digital calliper (Digitronic Calliper, 144–15 D (Wilson & Wolpert, Utrecht, The Netherlands)) while the animals were under general anaesthesia at days 0 and 35. All the measurements were carried out twice by two investigators independently within a period of few minutes and the reliability of the measurements was assessed by using the intraclass correlation coefficient (ICC) as described in previous studies^11,42^. Tooth movement was calculated by subtracting the distance between the teeth on day 35 from the distance between the teeth on day 0.

### Preparation of histology samples and bone histomorphometry

**Figure 3:**
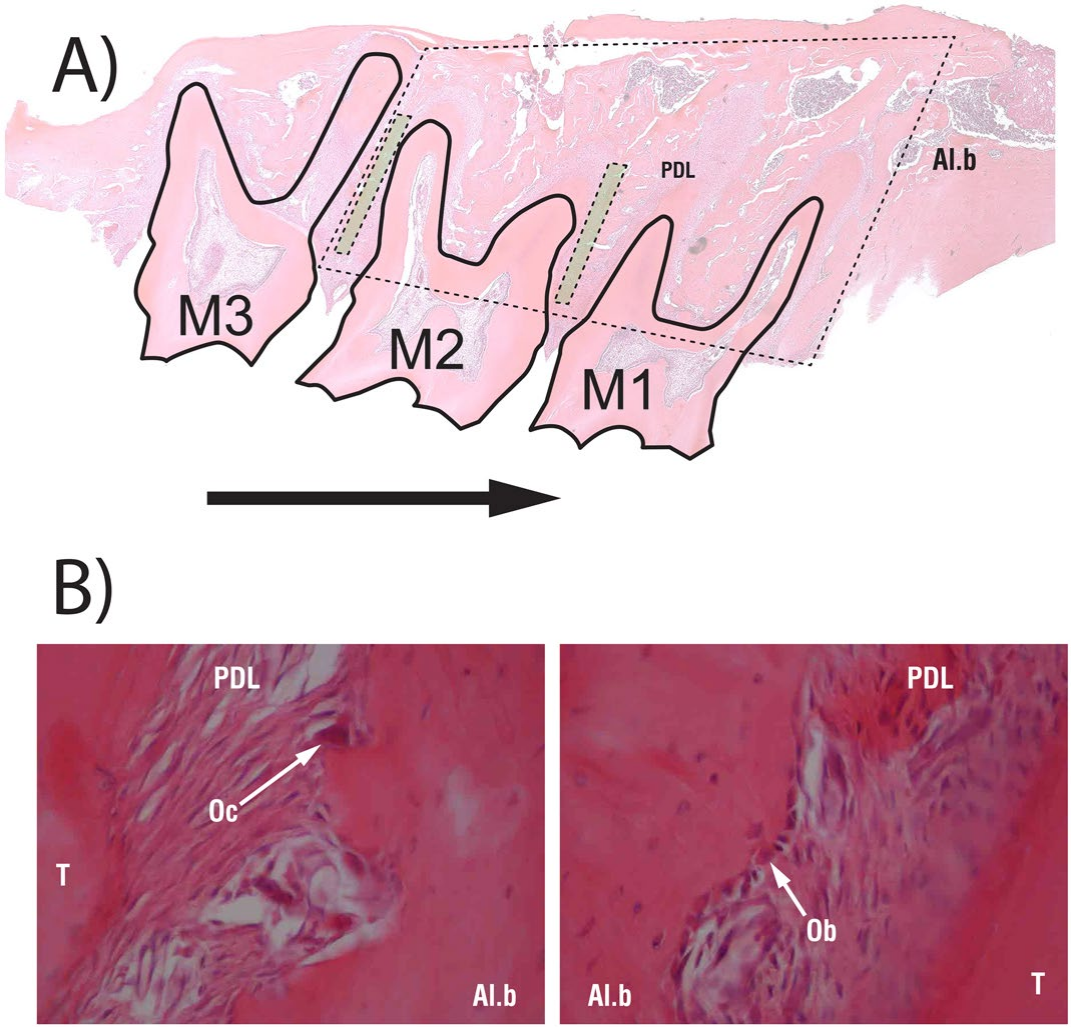
(**A**) Schematic representation of the examined areas in part of the maxilla with all three molars (M1, M2, M3). Alveolar bone volume was determined around the first and second maxillary molars (area surrounded by dashed line). Osteoblast and osteoclast volumes were determined along the mesial and distal roots of the second maxillary molar (green areas surrounded by dashed line). The arrow shows direction of tooth movement. (**B**) Examples of osteoclast (left picture) and osteoblast (right picture) under 40-fold magnification. Al.b—alveolar bone; Ob—osteoblast; Oc—osteoclast; PDL—periodontal ligament; T—tooth.

On day 35, all the animals were sacrificed by intraperitoneal injection of anaesthetics and carbon dioxide. Tissue samples of the maxilla containing all 3 molars were taken. Samples were prepared in vertical section perpendicular to the occlusal plane of the molars. Paraffin blocks were then prepared and stained with haematoxylin and eosin (Fig. 3) as previously published in Plut et al.^42^.

Bone histomorphometry was used to determine alveolar bone, osteoclast and osteoblast volume density in all four groups. Histomorphometry was performed by using a point-counting method. For this purpose, the stereologic cycloid grid system incorporated into the ocular of a light microscope (BX-60, Olympus, Tokyo, Japan) was used. As described by Sprogar et al.^11^, the alveolar bone area was expressed as the percentage of alveolar bone area versus the tissue area consisting of tooth, PDL, connective tissue and bone marrow spaces. In addition, the osteoblast and osteoclast areas were defined as the alveolar bone area covered with osteoblasts or osteoclasts versus alveolar bone area. The cells were counted in the alveolar bone alongside the mesial and distal roots of the second molar^42^. However, because 20 sections from each specimen were examined, alveolar bone area, osteoblast area and osteoclast area were extrapolated to alveolar bone volume, osteoblast volume and osteoclast volume, as we already described in previous studies^11,42^.

### Endothelin 1 (ET-1) determination

Endothelin 1 levels were measured in rat serum in all 4 groups using the commercially available kit Endothelin 1 ELISA Kit (ab133030, Abcam, Cambridge, MA, USA), according to the manufacturer’s instructions. Briefly, standards and samples were added to the designated wells of a microplate, precoated with an endothelin-1 specific antibody. The plate was incubated at room temperature for 1 h, washed and HRP-conjugated. The endothelin 1 detection antibody was added to each well. After incubation, the unbound detection antibodies were removed and TMB was added to visualise the HRP enzymatic reaction. After incubation, a stop solution was added and the absorbance was read at 450 nm, with correction at 570 nm, using Tecan Safire (Tecan Group Ltd., Switzerland).

### RNA isolation and semiquantitative RT-PCR

Osteocalcin and DMP1 gene expression levels were used to determine osteoblast activity, and the cathepsin K gene expression level was used to determine osteoclast activity in all 4 groups^43,44^. The maxillary bones with all 3 molars and their PDLs were excised and immediately frozen in liquid nitrogen. RNA isolation and semiquantitative RT-PCR was performed as described by Plut et al.^42^. Oligonucleotides for cathepsin K, osteocalcin and DMP1 were chosen from predesigned assays (TaqMan Gene Expression Assays, Applied Biosystems). Thermal cycling, construction of standard curve and cDNA amplification and quantification were performed as we reported in a previous study^43^. In order to exclude variations from different inputs of total mRNA to the reaction, data on cathepsin K, osteocalcin and DMP1 were normalised to an internal housekeeping gene, GAPDH, for which data was obtained by using TaqMan GAPDH predesigned assays (TaqMan Gene Expression Assays, Applied Biosystems). All the reactions for standard samples and for samples from all 4 groups were performed in duplicate. The data were averaged from the values obtained in each reaction^43^. For all the transcripts tested, a time-course-dependent gene expression consensus profile was observed after normalisation to the expression of the housekeeping gene GAPDH.

### Statistical analyses

The data were expressed as means ± standard error of the mean (SEM) and calculated for each parameter for all the animal groups. The evaluated parameters were tooth movement, alveolar bone volume density, osteoclast and osteoblast volume densities, serum ET-1 level, and gene expression levels of cathepsin K, DMP1 and osteocalcin. Comparisons within and between the groups were performed using analysis of variance (ANOVA), followed by the Tukey multiple comparison test. A P value less than 0.05 was considered statistically significant.

Interexaminer reliability for the measurements of the distance between the teeth was tested using the intraclass correlation coefficient (ICC).

In the results, not all the groups contained the initial number of rats (n = 8 per group). During the experiment some of the rats had to be excluded. The number of samples in each group is explicitly stated in the Figures.

## Results

### Orthodontic tooth movement and physiologic distal drift measurements after 35 days

**Figure 4:**
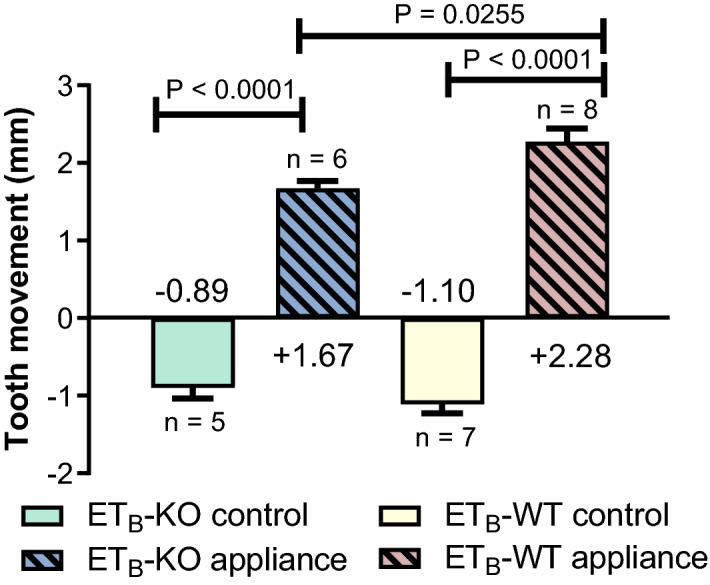
Amount of total tooth movement after 35 days. Significant differences were observed between the ET_B_-KO and ET_B_-WT appliance groups (P = 0.0255) and between appliance and control groups (P < 0.0001). The physiological distal drift was non-significantly decreased in the ET_B_-KO control group in comparison to the ET_B_-WT control group. The data are presented as mean ± SEM and analysed by the one-way ANOVA and Tukey post hoc tests.

The overall mean value of the ICC for all the measurements of the distances was 0.938. In the ET_B_-KO appliance group the amount of OTM (1.67 mm ± 0.10 mm) was significantly less pronounced than in the ET_B_-WT appliance group (2.28 mm ± 0.17 mm) on day 35 (P = 0.0255). The physiologic distal drift did not significantly differ between the ET_B_-KO and ET_B_-WT groups (Fig. 4).

### Histomorphometric analyses

**Figure 5:**
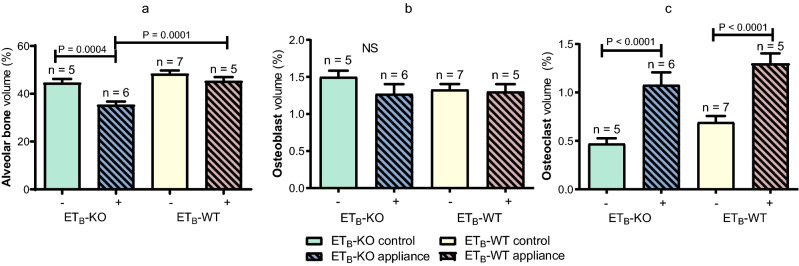
Histomorphometric analyses of the maxillary bone specimens after 35 days. The alveolar bone volume in the ET_B_-KO appliance group was significantly less than in the ET_B_-WT appliance group (P = 0.0001) (**a**); no significant (NS) differences in the osteoblast volume were observed between the groups (**b**); the osteoclast volume was significantly increased in the ET_B_-KO and ET_B_-WT appliance group compared to the control groups (P < 0.0001) (**c**). The data are presented as mean ± SEM and analysed by the one-way ANOVA and Tukey post hoc tests. (−)—control, ( +)—appliance.

The histomorphometric analysis showed that after 35 days of OTM the alveolar bone volume was significantly lower in the ET_B_-KO appliance group (35.26% ± 1.47%) in comparison to ET_B_-KO control group (44.45% ± 1.63%) (P = 0.0004). Furthermore, the alveolar bone volume was significantly less in the ET_B_-KO appliance group than in the ET_B_-WT appliance group (45.26% ± 1.74%) (P = 0.0001) (Fig. 5a). No significant differences in the osteoblast volume were observed between the groups. Osteoblast volume was non-significantly increased in the ET_B_-KO control group in comparison to the other three groups (Fig. 5b). The osteoclast volume was significantly increased in the ET_B_-KO (1.08% ± 0.13%) and ET_B_-WT appliance (1.30% ± 0.10%) groups compared to their controls (0.47% ± 0.06% and 0.69% ± 0.06%, respectively) (P < 0.0001). The osteoclast volumes in the ET_B_-KO appliance and the ET_B_-KO control groups were less than in the ET_B_-WT groups, but the differences were not significant (Fig. 5c).

### Serum endothelin 1 levels

**Figure 6:**
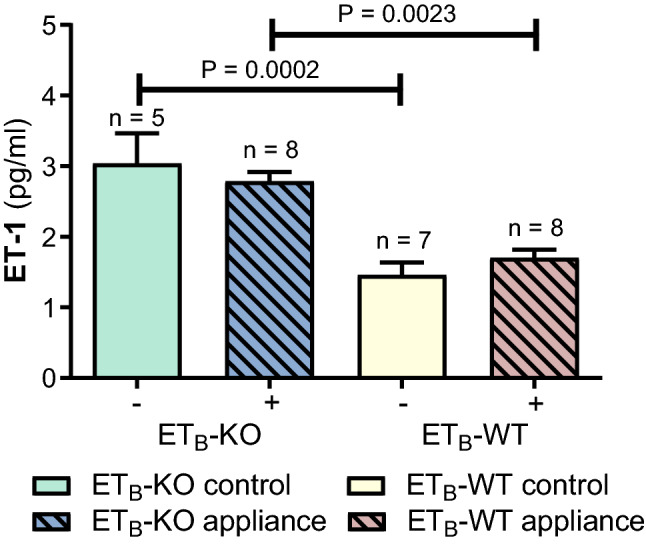
Blood serum endothelin level (ET-1) during the experimental period (35 days). The serum endothelin level (ET-1) was significantly higher in the ET_B_-KO control rats than in the WT control rats (P = 0.0002), and in the ET_B_-KO appliance group compared to the WT appliance group (P = 0.0023). The data are presented as mean ± SEM and analysed by the one-way ANOVA and Tukey post hoc tests. (−)—control; ( +)—appliance.

The serum endothelin level (ET-1) on day 35 was significantly higher in the ET_B_-KO control rats (3.04 pg/ml ± 0.43 pg/ml) compared to the WT control rats (1.46 pg/ml ± 0.18 pg/ml) (P = 0.0002) and between the ET_B_-KO appliance rats (2.78 pg/ml ± 0.13 pg/ml) and the ET_B_-WT appliance rats (1.70 pg/ml ± 0.12 pg/ml) (P = 0.0023) (Fig. 6).

### RT-PCR analysis

**Figure 7:**
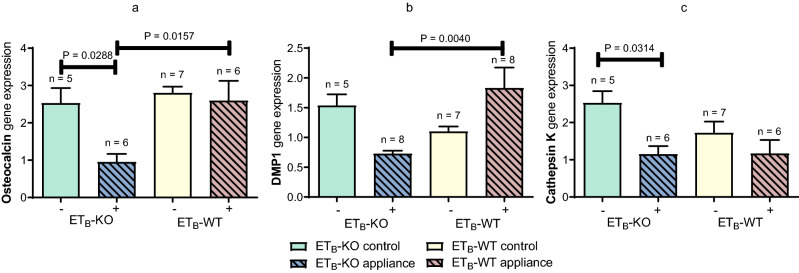
Relative gene expression levels of osteocalcin, DMP1 and cathepsin K in the alveolar bone and periodontal ligament after 35 days of OTM. Osteocalcin (**a**) and DMP1 (**b**) gene expression levels were significantly downregulated in the ET_B_-KO appliance group compared to the ET_B_-WT appliance group (P = 0.0157 and P = 0.0040, respectively). A significant difference was observed in the gene expression level of cathepsin K between the ET_B_-KO groups (P = 0.0314), but not between the ET_B_-WT groups (**c**). All gene expressions were normalised to the reference gene GAPDH. The data are presented as mean ± SEM and analysed by the one-way ANOVA and Tukey post hoc tests. (−)—control, ( +)—appliance.

The gene expression level of osteocalcin on day 35 was significantly downregulated in the ET_B_-KO appliance group compared to the ET_B_-WT appliance group (P = 0.0157). A significant difference in the gene expression level of osteocalcin was also determined between the ET_B_-KO appliance group and the ET_B_-KO control group (P = 0.0288) (Fig. 7a). Similarly, the gene expression of DMP1 was significantly downregulated in the ET_B_-KO appliance group in comparison to the WT appliance group (P = 0.0040) (Fig. 7b). The gene expression level of cathepsin K was downregulated in both appliance groups compared to the control groups. Significant differences were determined between the ET_B_-KO control group compared to the ET_B_-KO appliance group (P = 0.0314) (Fig. 7c).

## Discussion

The results of this study showed that ET_B_ is involved in bone modelling during the late stage of OTM in the rat animal model. The amount of OTM after 35 days of the experiment was significantly less in the ET_B_-KO appliance group than in the ET_B_-WT group (P = 0.0255). There was a significant difference in the alveolar bone volume in the ET_B_-KO appliance group compared to the ET_B_-WT appliance group (P = 0.0004), probably due to diminished osteoblast activity in the ET_B_-KO appliance group.

Some of the ET_B_ antagonists showed significant inhibition of ET-1 effects in vitro^45,46^; however, none of the available antagonists have an established pharmacological and toxicological profile. Furthermore, they have to be administered intravenously and daily intravenous application over a longer period of time represents a great deal of stress for the animals. To study the effects of ET-1 in a reduced ET_B_ response, the most suitable model is to use ET_B_ knock-out animals. The ET_B_-KO strain of rats used in the present study is described as a natural mutation in the progeny of a Wistar rat. Mutations in the ET_B_ gene have been linked to Hirschsprung’s disease in humans, a congenital disease characterised by aganglionic megacolon, an absence of enteric ganglia, and a lack of innervations to the lower gastrointestinal tract. The disease is associated with polymorphism and several missense mutations in the ET_B_ gene which lead to decreased expression, changes in cell signalling, and loss of endothelin ET receptor function^47,48^. The animals used in this study were incorporated with a transgene (DβH) to enable the development of a normal enteric nervous system. The resulting transgenic rats are healthy but present with a total absence of ET_B_ in all non-adrenergic tissues^40^. The animal model of OTM used in this study had already been confirmed as appropriate for studying the role of endothelin system in bone modelling^10–12^. The advantage of the model was the minimally invasive placement of the coil spring between the molars and incisors to maximally avoid injuries to the vital structures in the incisors and surrounding structures which may otherwise cause an inflammatory response and interfere with the results of the OTM. The force used in the experiment was constant and continuous^41,49^. The duration of the experiment is important in studying the processes in bone modelling during OTM. Bone formation on the tension side and bone resorption on the pressure side has been reported in the late stage of OTM; a time period usually around 2–4 weeks after the force has been applied^17^.

Both appliance groups showed lower alveolar bone volume compared to their control groups. Furthermore, the alveolar bone volume in the ET_B_-KO appliance group was significantly less than that of the ET_B_-WT appliance group. Differences in alveolar bone volume during physiological and pathological processes depend on the relationship between bone formation and bone resorption. Similarly to our study, histomorphometric analyses in previous studies have shown that alveolar bone volume was less in appliance groups than in their control groups^11,42,43^. We studied bone formation by determining osteoblast volume and osteoblast activity using osteocalcin and DMP1. There were no significant differences in osteoblast volumes between the four groups, but there was a significant decrease in osteoblast activity as determined by the gene expression levels for osteocalcin and DMP1 in the ET_B_-KO appliance group compared to the ET_B_-WT appliance group after 35 days of OTM. It has been shown that bone formation after the application of orthodontic force is increased predominantly by stimulating the differentiation of osteoblasts, and to a lesser extent by an increase in the number of these cells^50^. This is in concordance with our results, which show no considerable difference in osteoblast volume between the groups, but significant changes in osteoblast activity. Osteocalcin plays an important role in the maturation of mineral species^51^, and modulates osteogenic differentiation of MSCs^52^; DMP1 has a similar role in osteoblast differentiation and matrix mineralisation. Interestingly, it has also been reported that recombinant DMP1 induces the osteogenic differentiation of human periodontal ligament cells^53^, and appears to play an important role in the osteogenic differentiation of dental follicle stem cells^54^.

**Figure 8:**
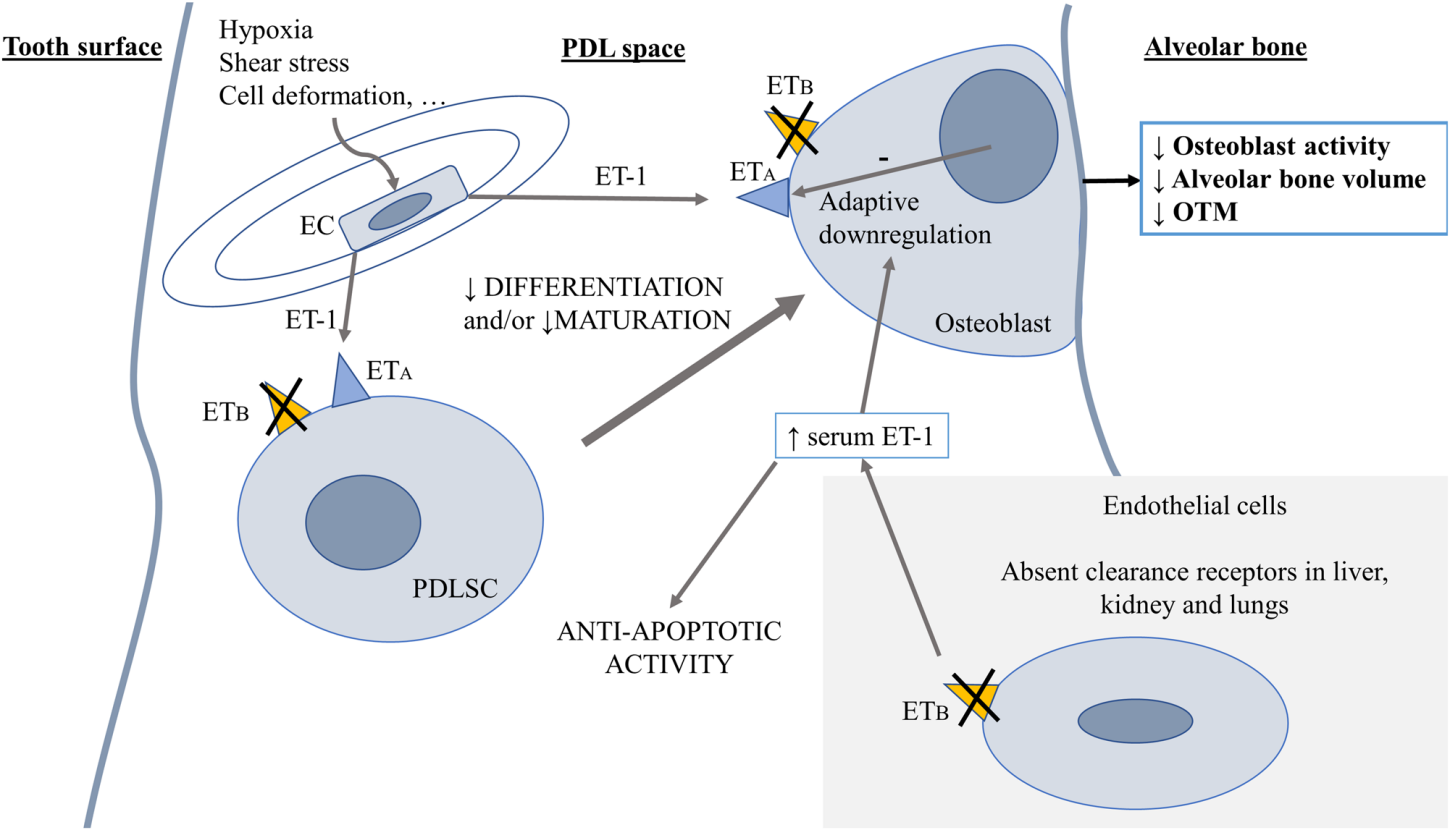
Possible mechanisms in PDL space are represented in this Figure, ultimately leading to decreased alveolar bone volume and a lower amount of OTM in the ET_B_-KO rats. The crossed-out triangles indicate the absence of ET_B_ receptors. ET-1—Endothelin-1; ET_A_—Endothelin receptor A; ET_B_—Endothelin receptor B; EC—Endothelial cell; PDLSC—Periodontal Ligament Stem Cell.

In a study on Bone Marrow-Derived Stem Cells (BMSCs) it was shown, using specific antagonists, that both receptors ET_A_ and ET_B_ are involved in the differentiation of BMSCs into active osteoblasts, and the osteogenesis of BMSCs was attenuated by blocking ET_A_ and/or ET_B_ receptors. The findings of this study reveal that both ET_A_ and ET_B_ receptors and downstream AKT and ERK signalling are involved in ET-1 primed lineage specification of MSCs^24^. Similarly, a study on ET_B_-KO mice showed less fibroblast activation and myofibroblast formation in response to bleomycin or ET-1^25^. Therefore, in our study, the lower expression levels of osteoblast activity markers, DMP1 and osteocalcin could be a result of attenuated osteoblast maturation and/or osteogenic differentiation of MSCs, a process that is mediated through both receptors ET_A_ and ET_B_. In the absence of ET_B_ in the ET_B_-KO appliance group, osteogenesis is attenuated, resulting in a lower alveolar bone volume and a decreased amount of OTM (Fig. 8).

The bone resorption process was studied by determining osteoclast volume and osteoclast activity using cathepsin K, a marker of bone resorption. A considerable increase in osteoclast volume was determined in both appliance groups compared to the control groups after 35 days of OTM. Similar results were obtained in a previous study, where osteoclast volume increased in all animal appliance groups^11^. In the late stage of OTM, we expected the upregulation of the cathepsin K gene expression level in the ET_B_-KO and ET_B_-WT appliance groups, whereas the osteoclast volume increased significantly in both appliance groups^43^. However, a significant downregulation of the gene expression level of cathepsin K appeared in the ET_B_-KO appliance group compared to ET_B_-KO control group. Several lines of evidence suggest that changes in osteoclast function and volume/number do not always happen simultaneously^55^. In a previous study it was shown that the absence of ET_B_ in osteoclast specific ET_B_-KO mice impairs the formation of mature osteoclasts and impairs bone resorption activity with no influence on the expression of osteoclastogenic genes^56^. ET-1/ET_B_ axis was shown to enhance osteoclast differentiation via co-stimulation of RANKL and M-CSF signalling and ET_B_ deficiency impaired bone resorption activity and formation of mature osteoclasts^56^. However, in the present study, there were no significant differences between the ET_B_-KO and ET_B_-WT appliance groups in terms of osteoclast volume and osteoclast activity. The applied force due to the coil spring used in the OTM model induced osteoclast activity independently of the presence or absence of ET_B_ or increased circulatory ET-1.

In the present study, the amount of OTM in the ET_B_-KO appliance group was significantly less than in the ET_B_-WT appliance group. Because of the diminished alveolar bone volume and lower osteoblast activity, a higher amount of OTM would initially be expected. However, we observed a lower amount of OTM, and similar results were also reported in Plut et al.^42^. During OTM, due to the lower osteoblast activity, bone formation at the tension site cannot keep up with bone resorption at the compression site, and the consequences are a widening of the PDL space and increased tooth mobility^57^. Tooth movement requires a coupling of bone resorption and bone formation. Due to the delayed bone formation in the absence of ET_B_ there may be a further aggravation of the uncoupling of bone formation and bone resorption, expressing as suppressed bone turnover and resulting in lower amount of OTM. In rats, molars naturally drift distally. Therefore, there is predominately bone resorption on the distal side and bone formation on the mesial side of molar roots^58^. In the present study, the distal drift was smaller in the ET_B_-KO control group than in the ET_B_-WT control group, but there was no significant difference. These results suggest a different mechanism of alveolar bone turnover under physiological conditions in comparison to OTM, shown in the ET_B_-KO groups.

In the ET_B_-KO control group the serum ET-1 level was significantly higher than in the ET_B_-WT control group. It was also significantly elevated in the ET_B_-KO appliance group in comparison to ET_B_-WT appliance group. This is in concordance with many studies that confirmed the role of ET_B_ as a ET-1 clearance receptor^32,37–39,59^. The lower amount of OTM in this study could be assigned to several processes, among them attenuated osteoblast maturation and increased ET-1 due to a lack of circulatory ET-1 clearance. High ET-1 levels are normally almost exclusively cleared by endothelial ET_B,_ which was lacking in our animal model. It has been shown that chronic exposure of ET receptors to increased plasma ET-1 levels results in a significantly reduced density of ET_A_ in mice aortas, correlating with a reduction in functional response to ET-1^60^. Similarly, ET_B_-KO mice were found to have a 45% lower ET_A_ density and significantly reduced expression (lower ET_A_ mRNA) in peripheral tissues with no change in receptor affinity. A potential mechanism of reduced ET_A_ density in ET_B_-KO models was proposed as the compensatory downregulation of the receptor in response to higher circulating ET-1 levels as a result of a lack of ET_B_ clearing receptors or some other role of ET_B_ in the development of cells expressing ET_A_ receptors^60,61^. It is therefore possible that in our study high serum ET-1 in the ET_B_-KO groups resulted in downregulation of the ET_A_ receptors, which already have an established role in bone modelling, resulting in diminished alveolar bone volume and lower amount of OTM (Fig. 8).

Many studies report interactions (cross talk) between ET_A_ and ET_B_ receptors, which means that the activation or inhibition of one receptor subtype can alter the function of another receptor subtype. For example, using ET_B_ receptor-deficient rats it was reported that both ET_A_ and ET_B_ are involved in ET-1-induced DNA synthesis in astrocytes, accompanied by MAPK activation^62^. Similarly, it was shown that both ET_A_ and ET_B_ regulate lung myofibroblast proliferation, indicating possible interactions between receptor subtypes^63^. In addition, endothelin receptors (ET_A_ and ET_B_) also exist in the form of homo- and heterodimers^64^, and for many receptor heterodimers, co-expression of both receptor subtypes is crucial for functional receptor activity, pharmacological proprieties, maturation, and proper cell-surface trafficking^65,66^. Another example of cooperation between receptor subtypes are HEK 293 cells which, transfected with both receptors, display a considerably prolonged increase in intracellular Ca^2+^ concentration in response to ET-1 or the selective ET_B_ receptor agonist Sarafotoxin 6c, in comparison to more transitory responses of cells transfected with either receptor subtype alone^67^. It is therefore possible that similar interactions between the two receptor subtypes are also necessary for normal bone modelling during the late stage of OTM.

Another potential explanation for reduced OTM is anti-apoptotic activity of elevated ET-1 in the two ET_B_-KO groups. Specifically, apoptosis of osteocytes is critical for osteoclast activation and resorption at the compression site of the tooth during OTM^68,69^. Moreover, in a study of OTM with micro-osteoperforations it was shown that the rate of OTM is increased by increased apoptosis and cell proliferation of PDL cells^70^. Therefore, the lower amount of OTM in the absence of ET_B_ could be a result of several different processes, including the anti-apoptotic activity of ET-1 on numerous cells in periodontal tissues. One limitation of the study, however, are only two time points in the experiment. Thus, no exact mechanism of the action can be elucidated. We rather propose possible mechanisms through which ETB-KO modulated bone modelling during OTM (Fig. 8).

## Conclusion

Our results showed for the first time that ET_B_ is involved in bone modelling in the late stage of OTM. ET_B_-KO resulted in lower osteoblast activity and therefore decreased alveolar bone volume and lower amount of OTM. This could be due to the role of ET_B_ in osteoblast maturation and/or the differentiation of mesenchymal precursor cells, the adaptive downregulation of ET_A_ as a response to high levels of circulating ET-1 or anti-apoptotic activity of ET-1. Further research is needed to explain the exact mechanism by which ET_B_ modulates bone modelling in OTM, and which of the proposed mechanisms is predominant.

## Data Availability

The datasets generated during and/or analysed during the current study are available from the corresponding author on reasonable request.
